# A prospective study of specimen eversion to lateral rectum and valgus resection for low rectal cancer

**DOI:** 10.3389/fsurg.2022.926227

**Published:** 2022-07-18

**Authors:** Long Qian, Xiaoxu Huang, Li Xu, Hao Chen, Tingting Cao, Song Wang, Can Luo, Yabin Xia

**Affiliations:** ^1^ Department of Gastrointestinal Surgery, The First Affiliated Yijishan Hospital of Wannan Medical College, Wuhu, China; ^2^ Department of Radiotherapy, The Second People’s Hospital of Wuhu, Wuhu, China; ^3^ Department of Radiotherapy, The First Affiliated Yijishan Hospital of Wannan Medical College, Wuhu, China

**Keywords:** laparoscope, reverse puncture device technique, specimen pulled out through the anus, low rectal cancer, psychological assessment

## Abstract

**Purpose:**

To investigate the safety and efficacy of a reverse puncture device (RPD) and specimen eversion of the rectum for resection in total laparoscopic proctectomy.

**Methods:**

In a prospective study from August 2019 to March 2021, 40 patients underwent a procedure with an RPD and specimen eversion of the rectum for total laparoscopic low rectal cancer resection, that is natural orifice specimen extraction surgery (NOSES), were included in the NOSES group. Forty patients in the control group underwent conventional laparoscopic radical resection for low rectal cancer and were included in the LAP group. Intraoperative- and postoperative-related indicators, recovery and inflammatory factors, quality of life (QOL) and mental health were compared.

**Results:**

All operations were successfully completed. Compared with the LAP group, the NOSES group showed better short-term outcomes, such as time to eating, postoperative pain, and especially postoperative incision-related complications. At the same time, postoperative inflammatory factor levels, psychological trauma, life-related anxiety and depression scores, and QOL were better in the NOSES group than in the LAP group.

**Conclusions:**

The application of an RPD and specimen eversion of the rectum for total laparoscopic low rectal cancer resection is a technically feasible and safe approach with a short-term curative effect.

## Introduction

Currently, the incidence of colorectal cancer is increasing, especially the incidence of low rectal cancer (1). The main treatment methods are still surgical resection and minimally invasive methods, as these methods continue to gradually develop. The position of the low rectum is low, and access is limited by the stenosis of the pelvic segment. Conventional laparoscopic radical rectal resection cannot ensure sufficient margins when closing the distal end and involves a small incision in the abdomen that renders the method not completely minimally invasive. Palanivelu et al. reported the first case of natural orifice specimen extraction surgery (NOSES), providing a new treatment strategy (2). This approach does not involve an abdominal incision, reducing trauma and preserving function. On this basis, we used a reverse puncture device (RPD) and performed specimen eversion of the rectum for total laparoscopic low rectal cancer resection, i.e., NOSES. There have been few reports but no multidimensional evaluations of psychological factors, quality of life (QOL) and prognosis. This article aimed to provide a clinical basis for this surgical approach.

Most patients with rectal cancer undergo surgery that severely affects their physical and mental functioning, has a severe impact on their health-related QOL and renders them unable to return to preoperative performance levels. In addition to enduring postoperative complications, pain, and a long hospital stay, the patient also suffers adverse effects on their spirit and QOL. This article not only followed up with patients regarding short-term postoperative complications but also analyzed the postoperative mental health of patients through a professional psychological assessment scale. Thus, a new biopsychosocial therapeutic strategy was applied. The self-rating anxiety scale (SAS), the self-rating depression scale (SDS), and the short-list of QOL were used to evaluate the patients and their health after surgery, creating a foundation for further research in the future.

## Materials and methods

### General information

In all, 80 patients with rectal tumors who were treated by specific surgeons in the same treatment group at the First Affiliated Hospital of Wannan Medical College from August 2019 to March 2021 were included. Patients who underwent NOSES were included in the NOSES group, and those who underwent conventional laparoscopic surgery were included in the LAP group. According to the digital table method, the patients were randomly divided into the two groups, with 40 patients in the observation group and 40 in the control group. This study was registered with the Chinese Clinical Registry (ChiCTR2100048061).

The inclusion criteria were as follows: patient age ranging from 38 to 76; BMI of 26.2 ± 2.4; rectal adenocarcinoma confirmed by preoperative colonoscopy and pathology, defined as low rectal cancer with the lower edge of the tumor less than 5 cm from the dentate line (3); clinical tumor stage T1-T3 (according to the American Joint Committee on Cancer (AJCC) 7th edition TNM tumor staging criteria); no lymph node metastasis; diameter <6 cm; good cardiopulmonary function; and no distant metastasis. See Table 1 for details.

**Table 1:** General information.

	Gender		Age	BMI	T Stage			Mean tumor diameter (cm)
	Male	Female			T1	T2	T3	
NOSES	22	18	65.8 ± 6.9	25.8 ± 3.4	10	21	9	2.4 ± 0.2
LAP	24	16	65.2 ± 7.2	26.5 ± 3.5	9	19	12	2.5 ± 0.3
*P*-value	0.821		0.705	0.367	0.748			0.083

The exclusion criteria were as follows: inability to tolerate surgery or major organ disorders; multiple primary colorectal cancers; prophylactic stoma or preoperative chemoradiotherapy; emergency surgery for acute intestinal obstruction, perforation, or bleeding; and inability to undergo resection at the same time or presence of lung, bone or liver metastases.

### Surgical methods

Before the operation, the risk and operation method were explained to the family members, and written informed consent was obtained. Venous blood was collected one day before the operation for CRP, IL-6, and TNF-α detection, and preoperative antibiotic prophylaxis and bowel preparation were routinely performed. After general anesthesia, the patient was placed in a head-high foot-plantar lithotomy position. A 1-cm incision above the umbilicus was made, and a Veress puncture needle was used to establish pneumoperitoneum. The intra-abdominal pressure was set at 12–15 mmHg (1 mmHg = 0.133 kPa). A 5-mm trocar was placed on each of the 2 transverse fingers on the medial side of the left anterior superior iliac spine, and the laparoscope and corresponding instruments were placed The location, size, and presence of metastasis of the tumor in the abdominal cavity determined the surgical approach. All operations followed the general principles of total mesenteric excision (TME) and functional preservation. The rectum was freed to the pelvic floor, and the posterior hiatal ligament was transected to the internal and external sphincter space; we performed sharp dissection down the retrorectal space to the rectosacral fascia and dissected the lateral rectal space to the edge of the levator hiatus; In this way, the first was to ensure that the eversion can be easily achieved, and the second was to ensure that the total mesorectal excision can be performed. This procedure was performed following the “aseptic tumor-free” principle, with complete removal of the tumor and surrounding affected tissue, adequate negative margins, thorough lymph node dissection and careful abdominal washing.

**Figure 1:**
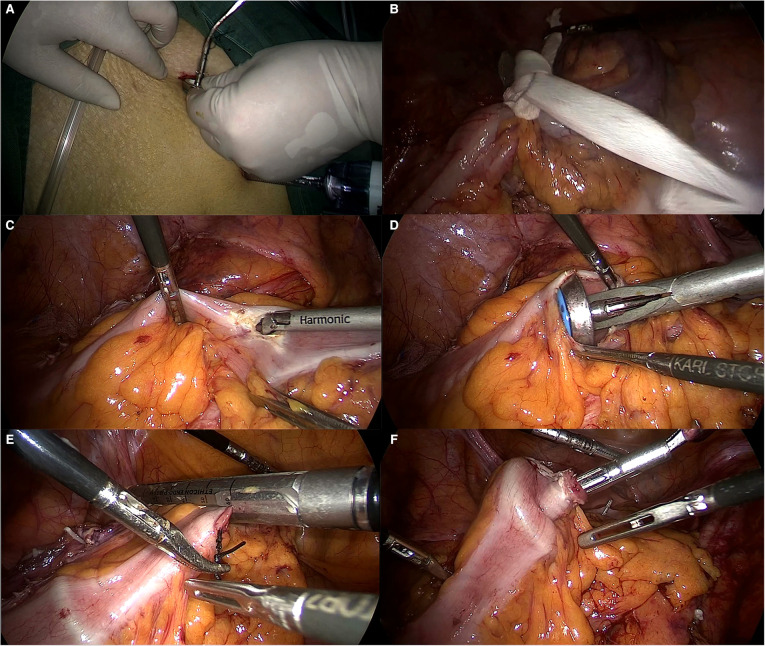
RPD technique. (**A**) The main operation hole is expanded, and the RPD is inserted with wire. (**B**) The proximal bowel is secured with string. (**C**) A longitudinal incision was made into the proximal bowel. (**D**) Intestinal placement of RPD (**E**) The silk suture was pulled against the anvil, and the precut line was used to disconnect the intestinal canal. (**F**) The anvil center rod was pulled out.

NOSES: The center rod of the anvil was placed in the left 12-mm trocar port (Figure 1A), and a 4-cm silk thread was inserted into the small hole at the tip of the connecting rod behind the anvil seat in advance and knotted for fixation. The proximal bowel was secured with string (Figure. 2B). A 2-inch bowel incision was made within 10 cm above the tumor (Figure. 2C). Sterilization with iodine was performed, and the highest point of the incision was located at the precut line; then, the abutment seat with the silk thread was placed into the intestinal canal completely, keeping a small gap where the silk thread protruded (Figure. 2D), and the incision was closed with a linear incision closure device (Figure. 2E). The silk thread was then used to pull out the anvil seat from the reserved intestinal canal space, thus completing the total laparoscopic insertion of the anvil seat (Figure. 2F). The assistant disinfected the perineum, fully expanded the anus, clamped the distal end of the rectum with oval forceps through the anus, and slowly dragged the tumor-bearing bowel and mesentery inversion through the anus. After repeated disinfection with iodophor, the bowel was rinsed with distilled water, and the cutoff rectum was closed with a Johnson & Johnson arc-cut stapler at a distance greater than or equal to 2 cm from the lower edge of the tumor. End-to-end colorectal anastomosis was performed by placing a 29-gauge circular stapler in the anus. Finally, the peritoneal cavity was lavaged with sterile saline. There was no auxiliary incision in the abdominal wall postoperatively (Supplementary Video S1).

LAP: The intestine was dissected 10 cm from the rectal tumor using a cutting device. A small incision was made in the middle of the abdomen. The tumor was excised outside the abdominal cavity, and a anvil seat was placed. The anus was inserted into the stapler to complete the enteroenteric anastomosis.

### Observational indicators

At 24 and 72 h after the operation, venous blood was collected for examination, and dynamic changes in inflammatory mediators were observed. During postoperative chemotherapy or telephone follow-up visits, the postoperative QOL and mental health of the patients were observed. Clinical indicators, including the visual analog scale (VAS), SAS, SDS, and QOL scores after treatment, as well as satisfaction with the surgical incision, were compared between the two groups of patients.

#### SAS

During the first chemotherapy period or one month after the operation, the patients were called back to complete the SAS questionnaire. The content filled in by the patient was used to calculate the corresponding score, which was multiplied by 1.25 to obtain the standard score for SAS status grading. Fifty points was used as the median of the judged scores, with lower scores indicating lower anxiety tendencies (4).

#### SDS

Depressive symptoms were defined as SAS scores ≥53 (53–62, mild; 63–72, moderate; >72, severe) according to a standardized scoring algorithm. The lower the score, the more severe the depressive symptoms (5).

#### VAS

Pain levels were assessed with the VAS at 1, 3, and 5 d after surgery, with 0–3 points indicating mild pain; 4–6 points, moderate pain; and 7–10 points, moderate pain.

#### QOL

According to the QOL scale, the QOL of the two groups of patients before and 6 months after treatment was evaluated, with a full score of 25 points; the lower the score, the worse the QOL (6).

#### Incision satisfaction

According to the scores of the homemade satisfaction questionnaire, the full score was 10 points, with 0–3 points indicating dissatisfaction, 4–6 points indicating basic satisfaction, and 7–10 points indicating great satisfaction. Satisfaction rate = (satisfaction + basic satisfaction) number of cases/total number of cases * 100%.

### Statistical analysis

All data were analyzed using GraphPad 8 statistical software. Measurement data conforming to a normal distribution are expressed as the mean ± standard deviation ($\bar{X}$ ± sd); independent-samples *t*-test was used for comparison between groups, and paired-samples *t*-test was used for comparison within groups. Count data are expressed as the number of cases/percentage (n/%) and were compared with the *χ*^2^ test. *P* < 0.05 was considered statistically significant.

## Results

### Intraoperative and postoperative general indicators

All operations were successful. In the NOSES group, the operation time was 132.1 ± 22.9 min, the screw insertion time (from cutting the tumor to the completion of anastomosis) was 8.5 ± 2.6 min, the intraoperative blood loss was 60.7 ± 47.5 ml, and there was no surgical auxiliary incision. The exhaust time was 2.4 ± 0.8 d, the hospitalization time was 7.1 ± 1.5 d, and the distance from the tumor to the stump was 2.3 ± 0.2 cm. No tumor infiltration was found in the postoperative pathological rectal stump. One case of anastomotic leakage that occurred in the NOSES group and two cases of anastomotic leakage that occurred in the LAP group were cured by abdominal irrigation combined with nutritional support; two cases of pulmonary infection that occurred in the LAP group were cured by anti-infective treatment. Four cases of incisional infection that occurred in the LAP group were cured after dressing changes and incision drainage. Significant advantages in terms of short-term postoperative complications, such as incisional infection, anastomotic leakage, pulmonary infection, anastomotic stenosis, and anastomotic bleeding, were observed. We followed up with the patients for 12–24 months after surgery, and no cases of tumor recurrence were found (Table 2).

**Table 2:** Intraoperative and postoperative observation indicators.

Characteristics	NOSES group (*N* = 40)	LAP group (*N* = 40)	*P*-value
Operation time (min)	182.1 ± 22.9	183.2 ± 25.5	0.839
Blood loss (ml)	60.7 ± 47.5	64.8 ± 49.1	0.705
Mean anvil placement time (hour)	8.5 ± 2.6	9.0 ± 4.8	0.564
Exhaust time (day)	2.4 ± 0.8	3.1 ± 1.3	0.005
Hospital stay (day)	7.1 ± 1.5	8.4 ± 1.2	<0.001
Utilization rate of analgesics after operation (cases (%))	3 (7.5%)	16 (40%)	0.001
Distance from tumor margin (cm)	2.3 ± 0.2	2.2 ± 0.3	0.083
Wexner score	6.8 ± 1.7	7.1 ± 1.8	0.446
Number of lymph nodes	13.8 ± 1.9	13.5 ± 0.9	0.370
Short-term complication rate(cases (%))	1(2.5%)	8(20%)	0.029

After 12 months of postoperative recovery, we used the Fecal Incontinence Severity Score (Wexner score) to evaluate the patients' postoperative anal function from the perspectives of formed stools, loose stools, gas, padding, and lifestyle changes, as the removal of specimens from the anus during NOSES may impair anal function. Our postoperative statistical analysis revealed no significant difference between the two surgical methods.

### VAS score

VAS scores were determined on the 1st, 3rd, and 5th days after the operation in the two groups. The pain in the NOSES group was better than that in the LAP group on the 1st and 3rd days after the operation (*P* < 0.05), and there was no difference between the two groups on the 5th day after the operation (Table 3).

**Table 3:** VAS score.

DAY	NOSES group	LAP group	*P*-value
1	3.61 ± 1.20	4.63 ± 1.42	0.001
3	2.18 ± 0.85	2.90 ± 1.37	0.006
5	0.69 ± 0.72	0.92 ± 0.65	0.138

### Inflammatory factors

Before surgery, there were no significant differences between the groups in terms of various inflammatory factors (all *P* > 0.05). At 24 and 72 h after the operation, the CRP, IL-6, and TNF-α levels were significantly different between the NOSES and LAP groups, with smaller magnitudes of change in the NOSES group (*P* < 0.05) (Table 4).

**Table 4:** Changes of inflammatory factors.

	Group	Preoperative	After 24 h	After 72 h
CRP (mg/l)	NOSES	2.09 ± 2.01	31.22 ± 7.11	131.24 ± 17.80
	LAP	1.87 ± 1.14	53.53 ± 11.31	187.21 ± 45.12
*P*-value		0.549	<0.001	<0.001
IL-6 (pg/ml)	NOSES	1.6 ± 0.8	136.0 ± 7.6	1293.2 ± 301.5
	LAP	1.7 ± 0.9	152.8 ± 12.4	1870.2 ± 217.4
*P*-value		0.601	<0.001	<0.001
TNF-α (pg/ml)	NOSES	10.2 ± 1.7	518.1 ± 71.0	446.3 ± 124.9
	LAP	10.5 ± 1.4	840.9 ± 68.8	696.4 ± 84.5
*P*-value		0.392	<0.001	<0.001

### SDS and SAS

The postoperative anxiety and depression scores of the patients were significantly lower in the NOSES group than in the LAP group (Table 5).

**Table 5:** SAS and SDS.

Group	SAS **score**	SDS **score**
NOSES	47.22 ± 7.36	45.36 ± 8.05
LAP	59.63 ± 8.44	52.47 ± 9.34
*P*-value	<0.001	0.001

### QOL

The postoperative QOL of the patients was better in the NOSES group than in the LAP group (*P* < 0.05), as shown in Table 6.

**Table 6:** Quality of life score.

Group	Mental function	Body Function	Material life
NOSES	54.9 ± 5.41	51.3 ± 7.24	60.8 ± 8.05
LAP	51.8 ± 5.64	46.9 ± 5.47	56.8 ± 4.44
*P*-value	0.014	0.003	0.007

### Comparison of satisfaction

Patients in the NOSES group were more satisfied with the incision after surgery (Table 7).

**Table 7:** Incision satisfaction.

Group	Satisfaction	Basically satisfaction	Satisfaction	Overall satisfaction
NOSES	22(55%)	12 (30%)	6 (15%)	34(85%)
LAP	14(35%)	10 (25%)	16 (40%)	24(60%)
*P-*value				0.023

## Discussion

The incidence of colorectal cancer continues to increase, and the search for better surgical methods continues. Compared with traditional open surgery, laparoscopic surgery has great advantages for lymph node dissection and blood vessel treatment because of the magnification effect of the laparoscopic lens. Since Fronklin et al first reported NOSES in 1993. In recent years, the NOSES procedure for collecting specimens from the vagina or anus has been greatly developed (7–9). NOSES reduces the need for auxiliary small incisions in surgery, reduces postoperative pain and the incidence of incisional infection, and greatly enhances abdominal aesthetics (10, 11).

The reverse puncture technique has been widely used in gastrointestinal surgery. We have also performed extensive reports on this topic (12). Compared with conventional small-incision-assisted laparoscopic colorectal surgery, the key technique in NOSES is inserting the anvil seat into the proximal bowel for anastomosis, which requires a surgeon with high technical skills. The traditional method involves manual purse-string suturing, but the requirements for laparoscopic suture technology are extremely high, and the operation time is long. The reverse puncture method was first used in gastric tract surgery (13) and then widely used in colorectal surgery (14). In NOSES, the principle of aseptic and tumor-free surgery has always been the focus of attention. Therefore, comprehensive patient evaluation and selection before surgery were crucial. Izquierdo et al. (15) reported that the NOSES procedure was not suitable for obese patients with a BMI over 30.In the perioperative period, enhanced CT or MRI were used to adequately assess the tumor location and size. Typically, a tumor size larger than 6.5 cm in diameter is considered an exclusion criterion for NOSES. For low rectal cancer, All patients were discussed with MDT in our hospital before treatment. At a fixed time and place every week, the MDT secretary of our hospital submits an application. Relevant gastrointestinal tumor treatment experts, such as pathologists, radiologists, oncologists, gastrointestinal surgeons, etc. Discuss and decide on a treatment plan. For patients with tumors whose clinical stage is T3, neoadjuvant chemoradiotherapy is required according to relevant guidelines (16). However, due to family reasons and the patient's own factors requiring surgical treatment, the patient's medical compliance was not enough. Therefore, for these patients, we did not perform neoadjuvant chemoradiotherapy and directly operated, this part of the patients was also included in our study. For female patients, vaginal removal is also an option. A reported study showed that NOSES performed with vaginal tumor removal did not increase the likelihood of fistulas (2) or affect postoperative sexual function (17). However, specimen removal through the anus is more physiological.

Our data indicated no differences in intraoperative blood loss, number of lymph nodes dissected, circumferential incision margins, postoperative follow-up or postoperative anal function (*P* > 0.05). The number of postoperative lymph nodes dissected is a key factor in the quality of surgical completion. The number of lymph nodes dissected is directly related to the survival period of patients after surgery. The College of American Pathologists (CAP) requires a minimum of 12 lymph nodes to be examined. The number of surgically dissected lymph nodes in both groups met this requirement. Compared with the LAP group, the NOSES group showed a shorter postoperative gastrointestinal function recovery time, a shorter postoperative hospital stay, lower postoperative pain scores, and a lower requirement for additional analgesics, and patients in the NOSES group were less concerned about the appearance of the abdominal wall after surgery and reported a higher degree of satisfaction (all *P* < 0.05). Because the patients did not have a small abdominal incision after surgery, there was no neurovascular damage caused by the incision, and the postoperative pain of the patient was reduced (18). After surgery, the patients could ambulate earlier, accelerating the postoperative ventilation time. At the same time, patients no longer resisted early encouragement to cough, the occurrence of lung inflammation was reduced, and postoperative anxiety was eliminated. These factors can reduce the stress response of postoperative patients. Postoperative stay was reduced in NOSES group but it was still long. The patient can eat liquid food after recovery of gastrointestinal function without abdominal pain, bloating and fever, normal biochemical and inflammatory indicators, and good incision healing. Discharge can be handled at this time. But the following rehabilitation treatment and suture removal cannot be well treated in community hospitals. On the other hand, these patients were older. For safety reasons, the discharge time of patients was relatively conservative. These factors contributed to the prolonged hospital stay of our patients.

The closure device was obliquely closed when we used the reverse puncture technique to transect the proximal bowel, a stump angle was generated after anastomosis of the digestive tract. We found that most cases of leakage after reverse puncture were not due to anastomotic leakage but were caused by this stump angle formed after anastomosis; thus, it is necessary to place reinforcing sutures with barbed thread. In addition, a major advantage of reverse puncture is that the entire plane is flatter after the anvil seat is placed than after the small abdominal incision purse-string sutures are placed, and there is less tissue in the middle of the anvil seat, which reduces the incidence of anastomotic tissue ischemia and anastomotic leakage. In addition, the rectum should be fully freed during the operation to ensure that the distal bowel can be pulled out of the anus. If excessive tension is found during reconstruction of the digestive tract, the splenic area should be released. To follow the “tumor-free” principle, the specimens extracted through the anus were washed with a large amount of iodophor and distilled water, and the abdominal cavity was washed after the operation.

As an exogenous trauma, surgery will inevitably cause a stress response in the human body. We monitored the postoperative levels of inflammatory factors to determine the extent of the trauma to the body that was caused by the two surgical methods. CRP is the most sensitive and pronounced acute-phase protein after trauma or stress (19). IL-6, as a core cellular molecule, is a main mediator of immune activity and inflammatory responses (20). TNF-α can promote the adhesion of neutrophils to the endothelium and participate in the systemic inflammatory response (21). Therefore, we evaluated systemic inflammatory changes by observing these three indicators. We found that compared with LAP, NOSES resulted in smaller changes in various indicators, with a faster decline. Thus, we concluded that the reverse puncture technique has certain advantages in terms of controlling the inflammatory response in patients undergoing total laparoscopic low rectal resection.

Currently, the incidence of colorectal cancer is increasing, and most patients know little about postoperative recovery, which leads to a series of negative emotions after surgery. Several studies have shown that NOSES and postoperative rehabilitation treatment can promote the early recovery of patients (22, 23). This study showed that the SAS and SDS scores and postoperative rehabilitation indexes of patients in the NOSES group were better than those in the LAP group. Traditional laparoscopic-assisted colorectal cancer resection results in more postoperative scarring and a longer recovery time due to the auxiliary small incision in the abdomen. NOSES is less invasive and accelerates recovery, which can significantly improve the prognosis and QOL of patients. Studies have shown that NOSES has a better cosmetic effect, with only a few small trocar holes in the abdomen, which can significantly improve the patient's postoperative satisfaction with the surgery.

While removal of the rectum from the anus increases doubts about the risk of infection, Bucher et al. (24) demonstrated that this procedure does not increase the risk of infection. There have been articles and studies suggesting that the use of sterile specimen bags can prevent the occurrence and recurrence of tumors to the greatest extent (25). In this study, we also flushed the rectum with plenty of iodophor. Postoperative abdominal and pelvic drainage tubes were routinely placed, as reported in the literature, which can also prevent bacterial contamination (15, 26). Moreover, monitoring the body's inflammatory factors after surgery revealed that this surgical method caused less trauma, and None of the patients in our NOSES group developed a postoperative infection.

Although we are a prospective study, the number of patients is relatively small, and future multicenter studies are needed to provide more robust evidence. We only focused on the short-term effects of surgery, as the follow-up period was insufficient for long-term analysis. However, we are the first to compare the two surgical approaches using a psychological assessment. In general, the reverse puncture technique has obvious advantages over traditional laparoscopic surgery in patients undergoing total laparoscopic radical resection for rectal cancer because specimens are removed through a natural orifice.

## Conclusions

For low rectal cancer, the reverse puncture technique is not inferior to conventional laparoscopy in total laparoscopic colorectal cancer resection and shows advantages such as short-term efficacy. Additionally, this technique can effectively improve the QOL and mental health of patients. This technique is highly selective for patients and is suitable for low rectal cancer with small tumor size and early clinical tumor stage.

## Data Availability

The original contributions presented in the study are included in the article/**Supplementary Materials**, further inquiries can be directed to the corresponding author/s.
